# Supplementary figures and images for: Phenotyping the virulence of SARS-CoV-2 variants in hamsters by digital pathology and machine learning
Source: PLoS Pathog. 2023 Nov 7;19(11):e1011589. doi: 10.1371/journal.ppat.1011589 (PMC10656012; doi:10.1371/journal.ppat.1011589)

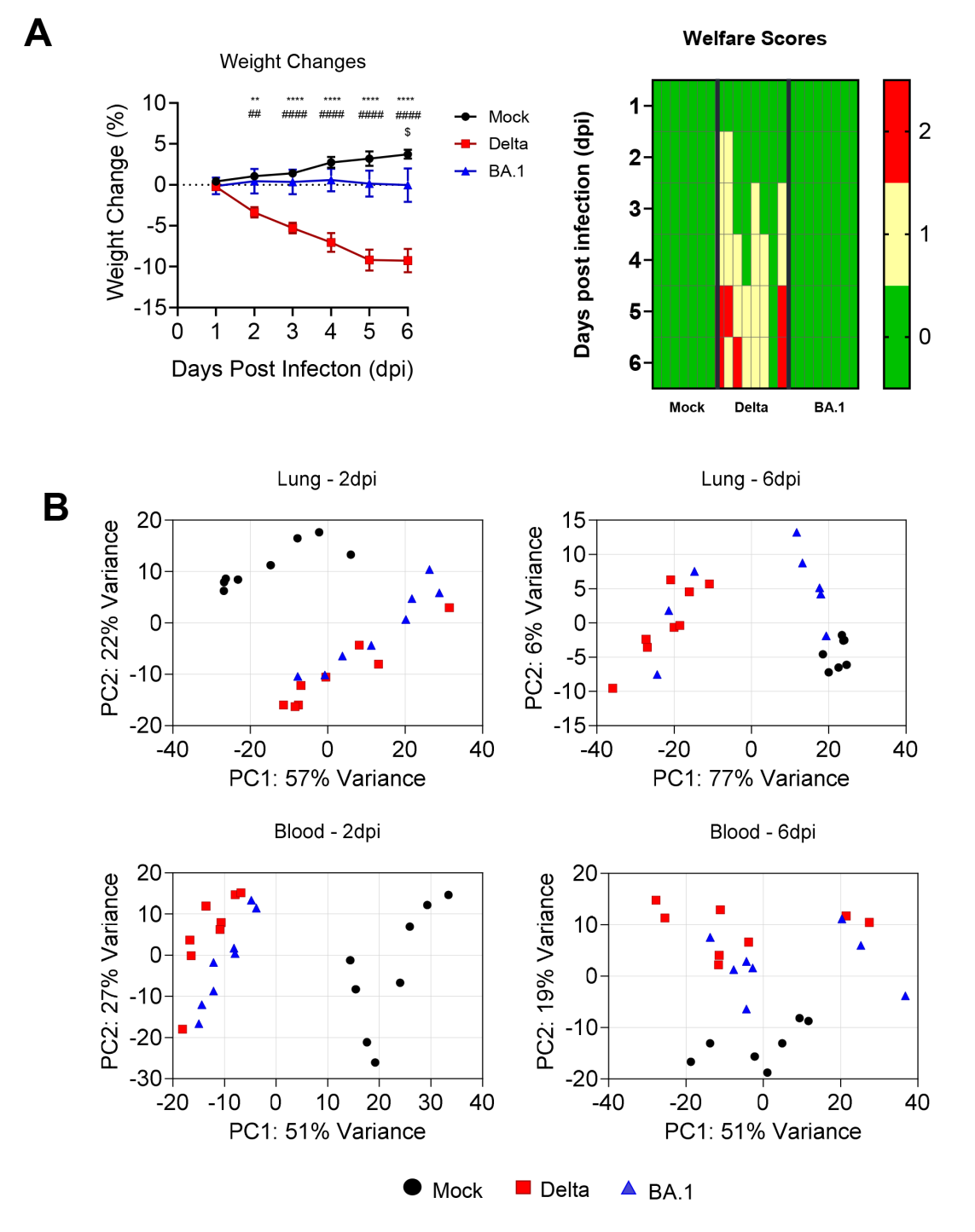

Supplement: S1 Fig — (A) Hamsters were infected with Delta or BA.1 intranasally. Mock-infected animals received media alone. The weights and disease scores of each animal was recorded daily. (B) All animals were culled 2- or 6-days post-infection (dpi), and RNA was extracted from lungs and blood for RNAseq. PCA plots indicate the variance between the different samples at 2 and 6 dpi. Data were obtained from n = 8 animals per group from two independent experiments (4 females and 4 males per group). (TIF) [file ppat.1011589.s001.tif]

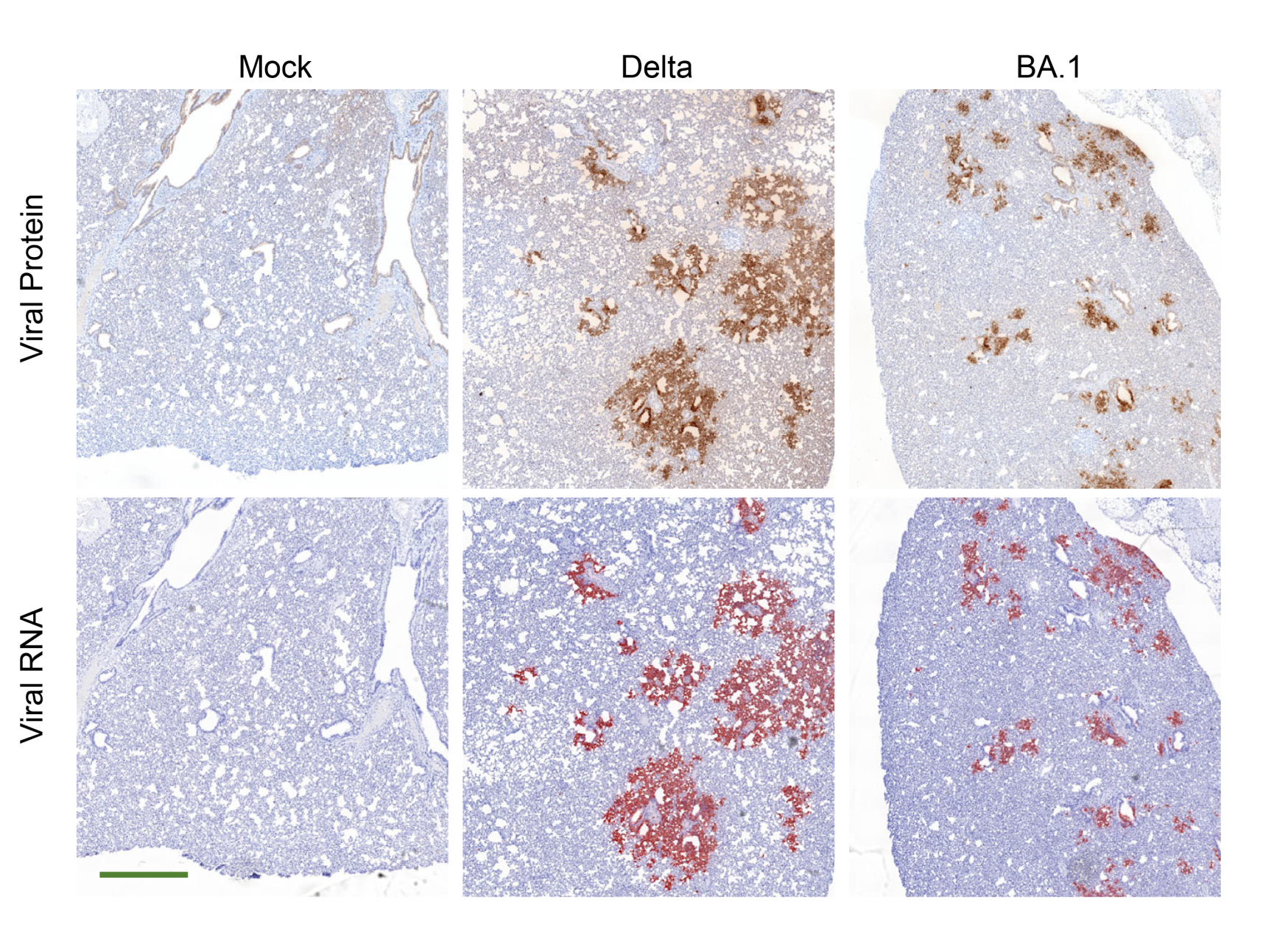

Supplement: S2 Fig — Micrographs of lung tissues collected from hamsters infected intranasally with either Delta or BA.1 (or mock-infected). Tissues were collected 2 days post-infection (2 dpi). Animals were culled at 2 dpi and lung sections were assessed for the presence of viral protein by immunohistochemistry, or viral RNA by in situ hybridisation. (TIF) [file ppat.1011589.s002.tif]

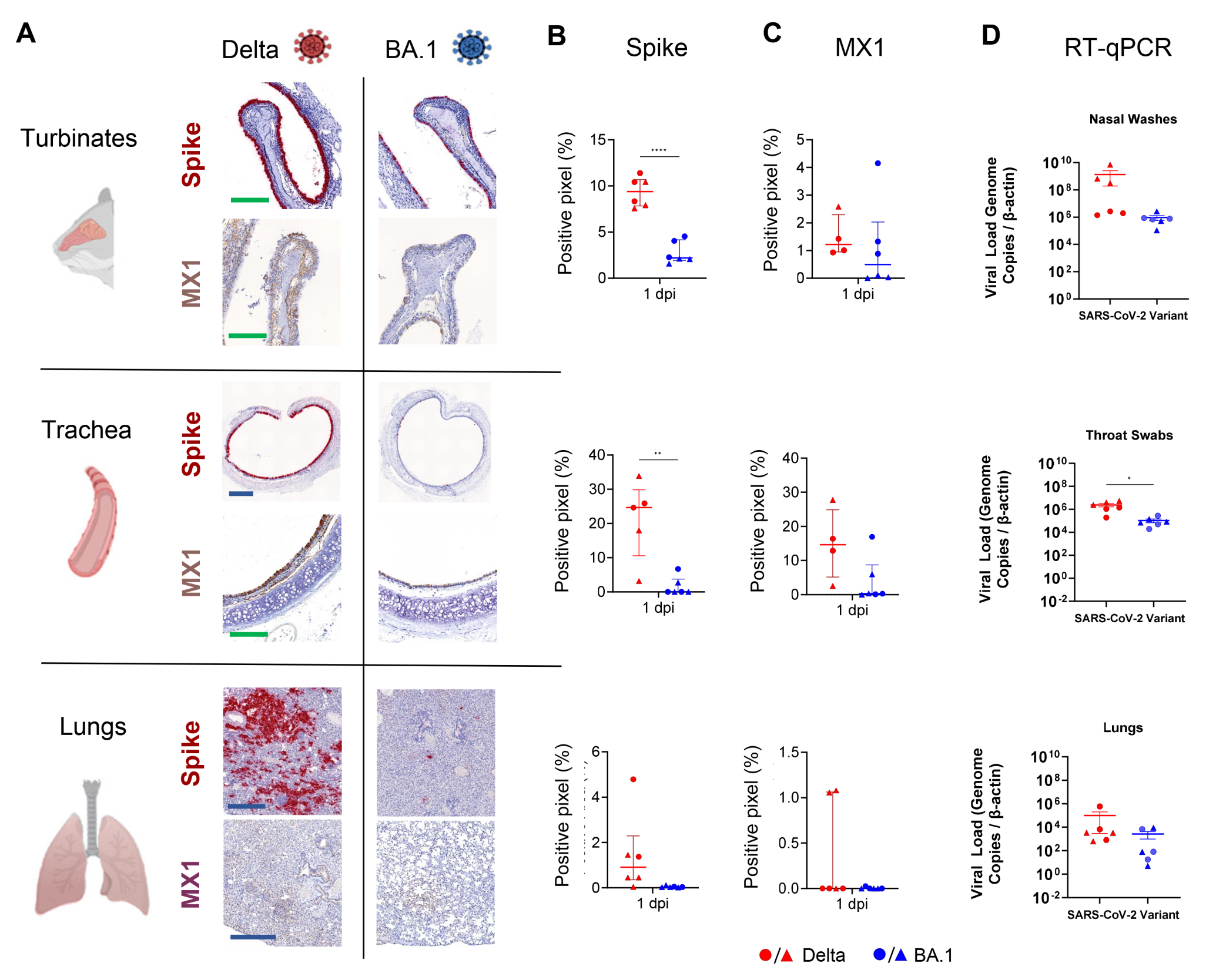

Supplement: S3 Fig — (A) Hamsters were infected with either Delta or BA.1 intranasally (or mock-infected). Animals were culled 1 day post infection and turbinates, trachea and lungs were collected for digital pathology analyses. (B) Tissues were assessed for the presence of spike RNA by in situ hybridisation or (C) for the expression of MX1 by immunohistochemistry. For signal quantification, slides were scanned with an Aperio VERSA 8 Brightfield, Fluorescence & FISH Digital Pathology Scanner (Leica Biosystems) at 200 x brightfield magnification. (D) Nasal washes, throat swabs and lungs were analysed for the presence of SARS-CoV-2 genomic RNA by RT-qPCR. Statistical analysis was performed using an unpaired t test, *<0.05, **<0.01, ****<0.0001. Data is representative of two independent experiments, n = 6 (3 females and 3 males per group). Males: triangles; females: circles. Blue scale bar: 500 μm; green scale bar: 200 μm. Graphics made using biorender.com. (TIF) [file ppat.1011589.s003.tif]

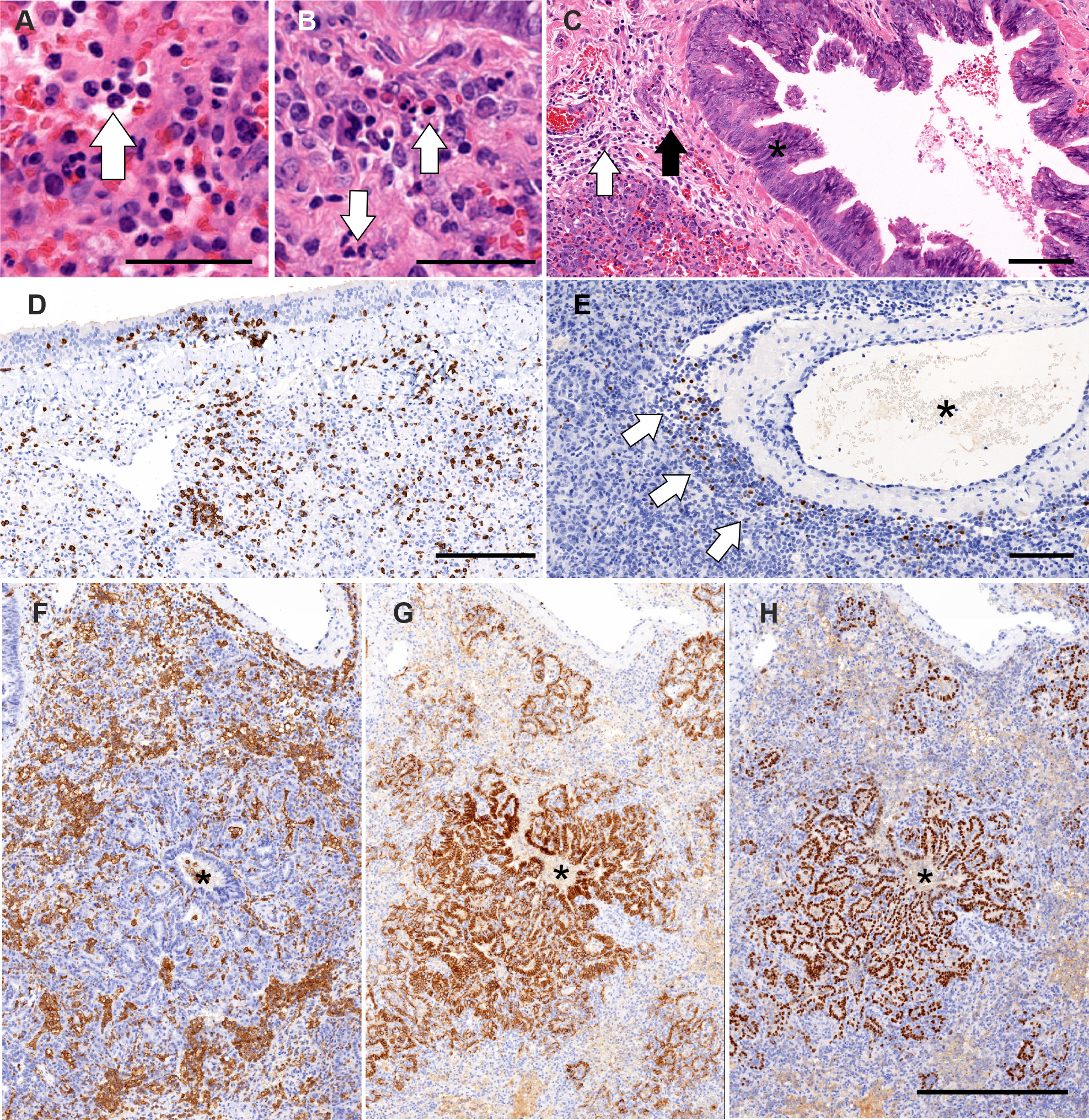

Supplement: S4 Fig — (A-B) Micrograph of lung sections stained with haematoxylin and eosin showing an infiltrate of neutrophils/heterophils (arrows, bars, 50 μm), and (C) lymphocytes (white arrow) and plasma cells (black arrow). The hyperplastic bronchial epithelium is shown with an asterisk (bar, 100 μm). (D) The diffuse distribution of lymphocytes is highlighted by immunohistochemistry by the presence of CD3+ T cells throughout the lung (bar, 200 μm), and (E) plasma cells (PAX5+) around the vessels (asterisk) (bar, 100 μm). (F-H) Micrographs of serial sections of lung of a Delta-infected hamster assessed by immunohistochemistry. Infiltrating macrophages are highlighted using by IBA1staining. Note the presence of macrophages surrounding an area with severe proliferation of epithelial cells arranged in rosette-like structure, as established by E-cadherin (G) and TTF1 staining (H). The asterisk highlights the centre of the proliferation in F-Hs (ars = 400 μm. (TIF) [file ppat.1011589.s004.tif]

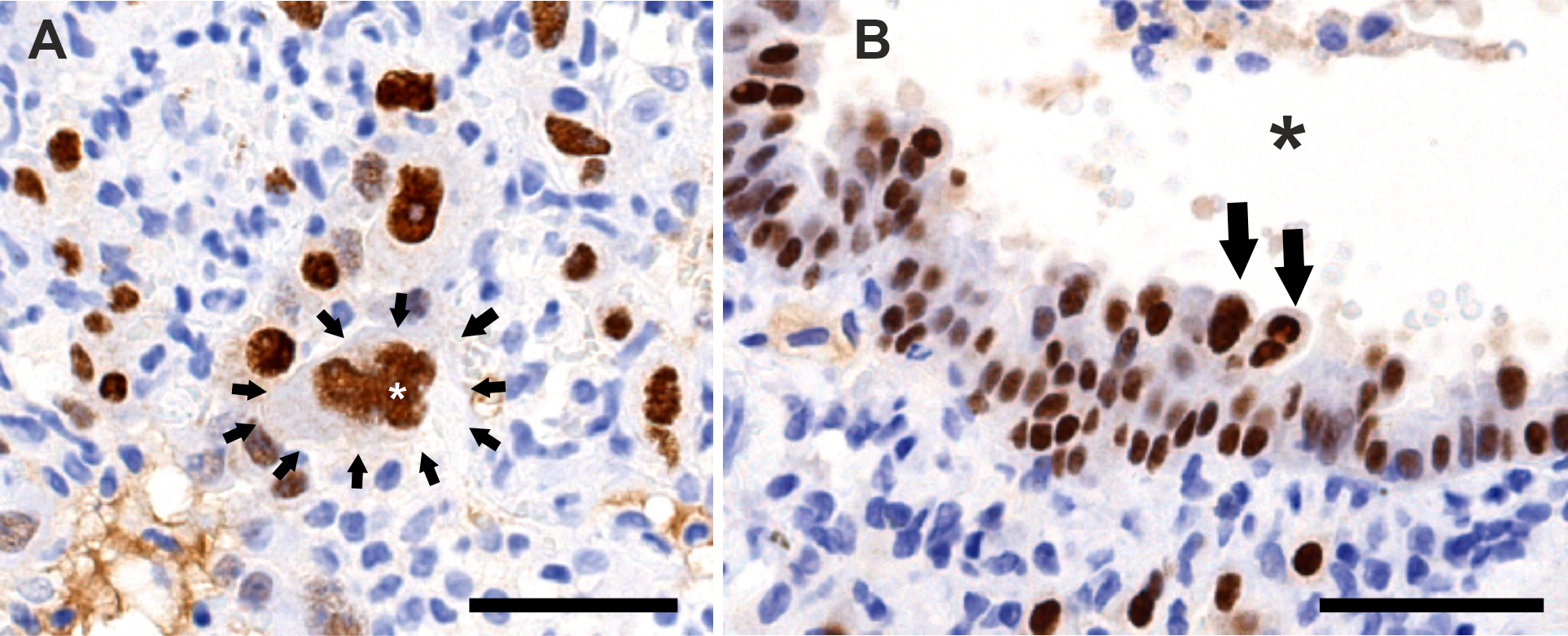

Supplement: S5 Fig — (A) Micrographs of lung sections analysed by immunohistochemistry using TTF1 antibodies. Within the consolidated lung parenchyma, the presence of cells with multiple, partially fused nuclei can be detected in some animals (arrows and asterisk). These cells are identified as syncytia (black arrows). (B) Also the bronchial epithelium shows in some cases multinucleated cells interpreted as syncytia (black arrows) on the luminal side of the bronchus (highlighted with an asterisk). A and B, TTF-1 staining; bars, 50 micrometres. (TIF) [file ppat.1011589.s005.tif]

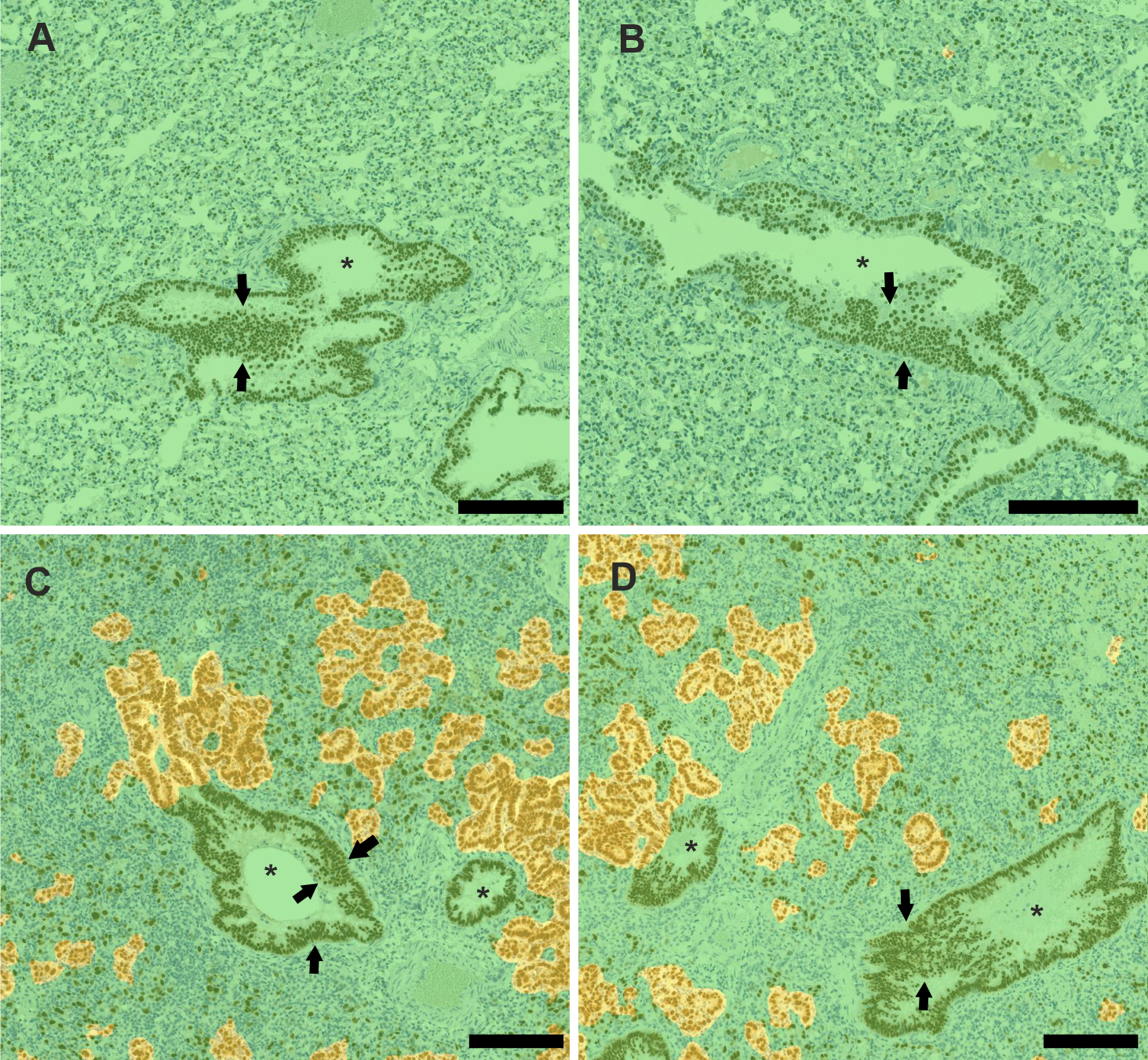

Supplement: S6 Fig — (A-B) Lung sections analysed by immunohistochemistry using TTF1 antibodies. TTF1+ cells include isolated type 2 pneumocytes and bronchial epithelial cells including artefacts of multilayered epithelium caused by sectioning (arrows). (C-D) Immunohistochemistry as in A-B analysed with HALO using the machine learning assisted module with the algorithm trained to ignore isolated type 2 pneumocytes and bronchial epithelium. Hyperplastic type 2 pneumocytes in rosette-like structures are highlighted in yellow by the software. Only these structures are quantified (shown in yellow). Asterisks highlight the lumen of bronchi. Bars, 200 micrometres. (TIF) [file ppat.1011589.s006.tif]

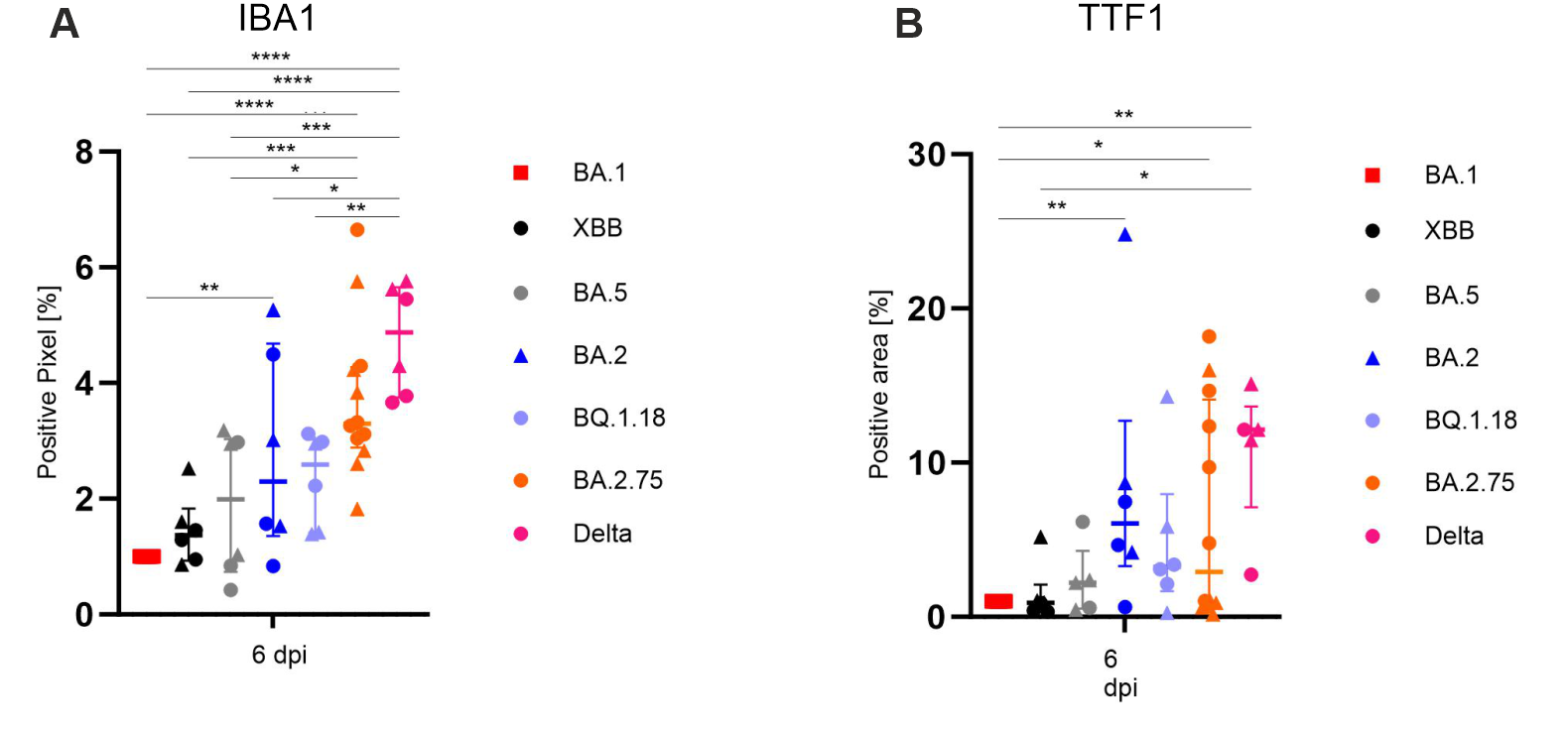

Supplement: S7 Fig — Data shown in Fig 6A, 6D, 6E, 6G and 6H was merged for either (A) IBA1- or (B) TTF-1-positive areas (type 2 pneumocyte hyperplasia) by normalising results to those obtained in BA.1-infected hamsters (normalised to 1). Statistical analysis was performed using a One-Way ANOVA with Tukey’s multiple comparisons test. Significance is indicated with *<0.05, **<0.01, ***<0.001, ****<0.0001. Male animals: triangles, female animals: circles. (TIF) [file ppat.1011589.s007.tif]

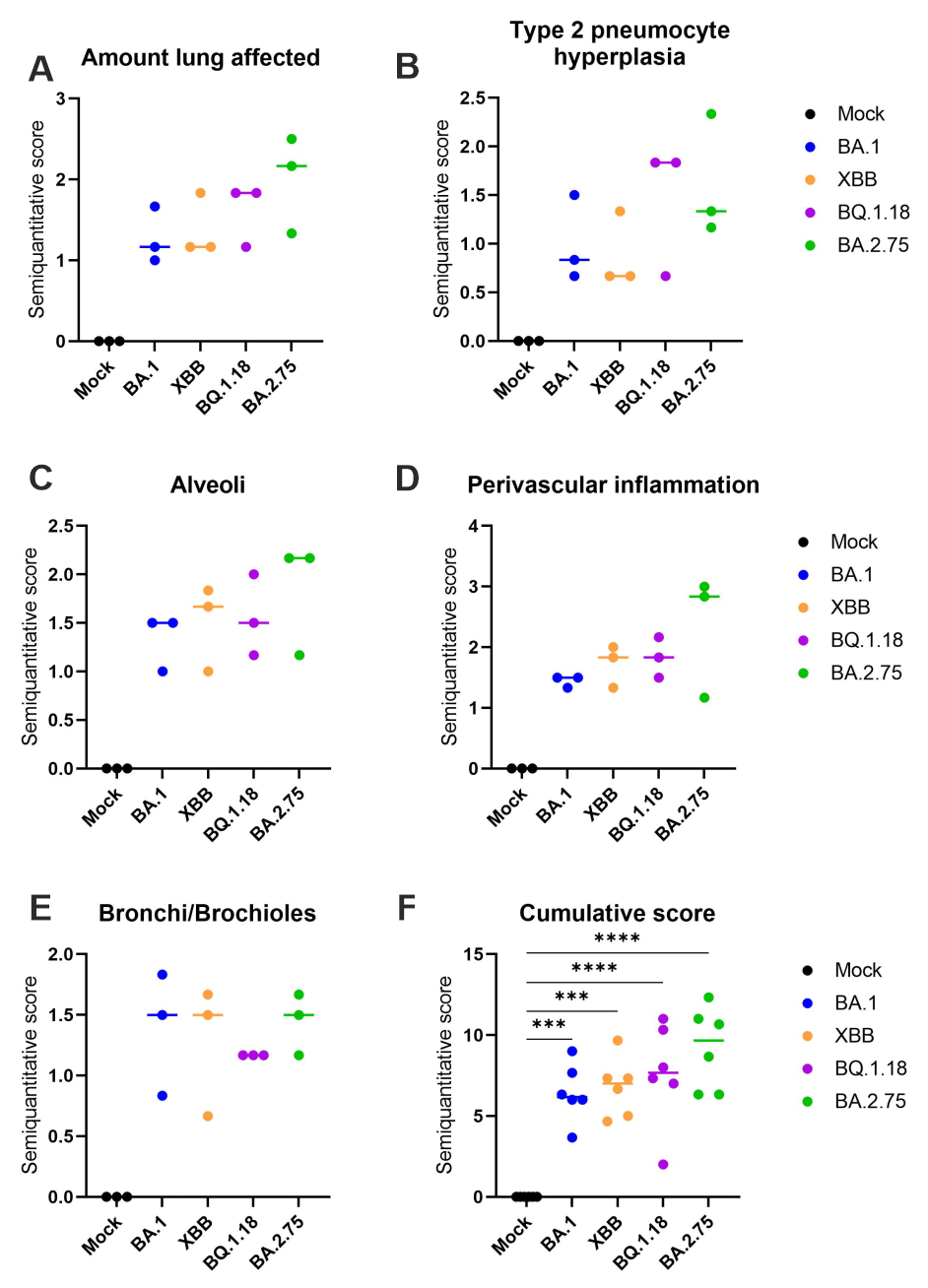

Supplement: S8 Fig — The amount of lung affected (A), respiratory epithelial cell or type II pneumocyte hyperplasia (B), alveolar inflammation (C), perivascular inflammation (D), bronchi/bronchioles inflammation (E) are shown as the average of the scores obtained in 6 animals per group. Each dot represents the value obtained by one Board-certified pathologist (not involved in the experimental phase of this study). The median is also highlighted. The final cumulative score (F) was calculated by adding per each animal the average scores by each pathologist (n = 6 hamsters per variant). Statistical analysis was performed using a One-Way ANOVA with Tukey’s multiple comparisons test. Significance is indicated with *<0.05, **<0.01, ***<0.001, ****<0.0001. (TIF) [file ppat.1011589.s008.tif]

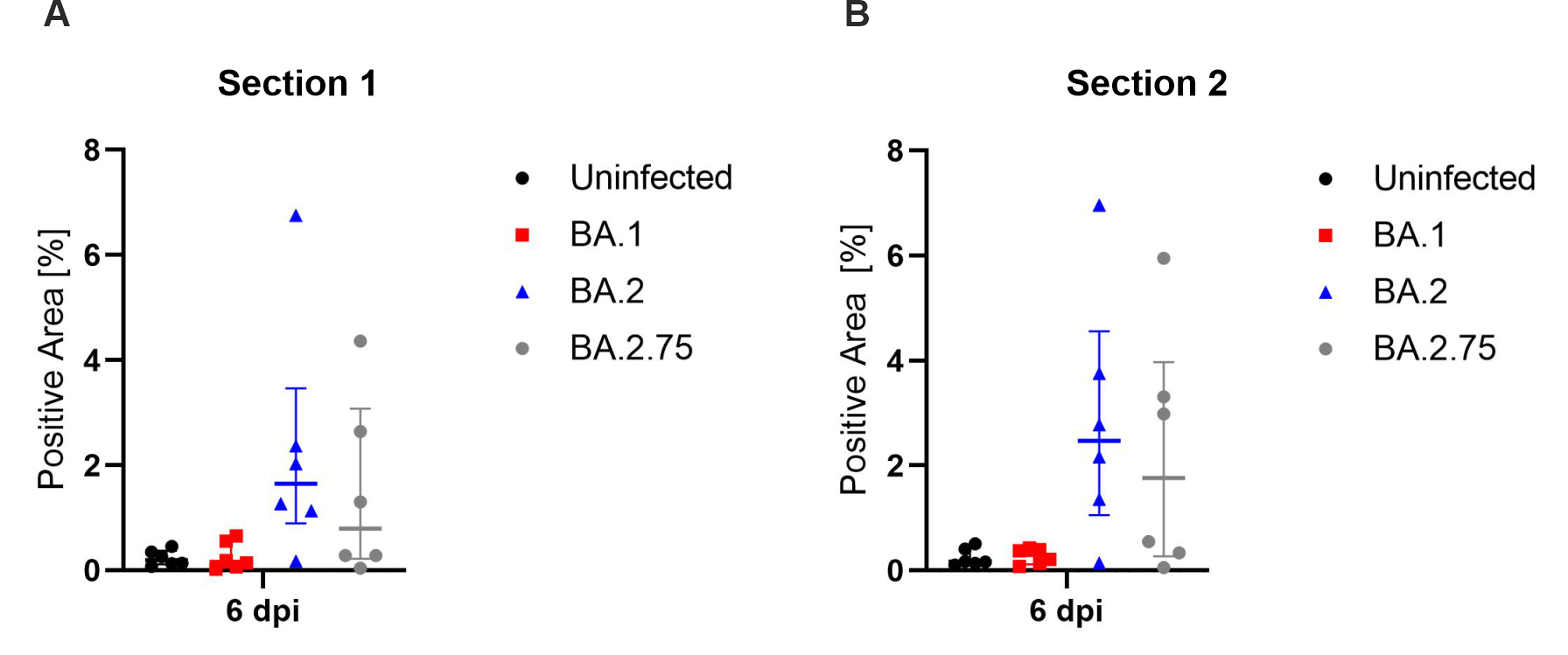

Supplement: S9 Fig — (A-B) Detection of alveolar epithelial hyperplasia (analysed as shown in S8 Fig) in hamsters experimentally infected with BA.1, BA.2 or BA.2.75. Data obtained in two distinct lung sections (Section 1 and 2) collected at approximately 100 μm from each other in the lung. Experiments have been processed, stained, scanned and analysed at different times. (TIF) [file ppat.1011589.s009.tif]
