# Supplementary material for: Phenotyping the virulence of SARS-CoV-2 variants in hamsters by digital pathology and machine learning
Source: PLoS Pathog. 2023 Nov 7;19(11):e1011589. doi: 10.1371/journal.ppat.1011589 (PMC10656012; doi:10.1371/journal.ppat.1011589)
Supplement: Attachment — Submitted filename: Rebuttal Letter.docx [file ppat.1011589.s010.docx]

Editors

PLoS Pathogens

October 2023

Re: Revision of manuscript PPATHOGENS-D-23-01272 “*Phenotyping the virulence of SARS-CoV-2 variants in hamsters by digital pathology and machine learning*” by Meehan, Herder *et al*

Dear Editors

First of all we would like to thank you and the Reviewers for editing the manuscript and providing useful feedback. We essentially agreed with all the key comments and made the necessary adjustment to the revised manuscript.

Overall, we have modified some of the main figures and added 5 new supplementary figures to clarify and address some of the reviewers’ suggestions. We have also made available in the Enlighten repository the algorithm and files used to measure epithelial hyperplasia (https://doi.org/10.5525/gla.researchdata.1513), and described in detail in the Methods section, the method used to quantify epithelial hyperplasia using machine learning approaches (see below).

In addition, please also note that we added new data related to one of the recently emerged SARS-CoV-2 variants, EG.5.1, to Figure 6. Data with this recent variant will further increase the impact of the manuscript.

Below, we have provided a detailed response to each of the points raised by the reviewers. We cut and pasted the reviewers’ points directly in this letter (in red) to facilitate the editorial process. Manuscript line numbers given in brackets refer to the plain version of the manuscript without tracked changes.

*Reviewer #1: Figure 4 - The authors should show either specific staining (preferred) or high resolution, highly magnified images to support their results text describing various neutrophil, lymphocyte and plasma cell infiltration and especially type 2 pneumocyte proliferation. These features cannot be observed from the H&E-stained sections that are shown.*

We thank the reviewer for this comment. In response to this we provide two additional supplemental figures, which include higher magnifications of the HE-stained sections (to show the neutrophils/heterophils and plasma cells in the lungs at 6 dpi, **Fig. S4 A - C**), and immunohistochemistry to show CD3-positive T cells and Pax5-positive plasma cells (**Fig. S4 D -E**). The type 2 pneumocyte proliferation is shown by serial sections stained with TTF-1 and E-cadherin to emphasise this point (**Fig. S4 F - H**). We also show multinucleated cells in **Fig. S5**.

We have modified the text accordingly to highlight these points and supplementary figures to the reader (see corrections in text lines 248-258 – note lines refer to clean final version of the paper).

“The authors should use scientific language to describe their phenotypic observations not use the word medusa on it’s own to describe lung pathology. While medusa is an apt comparison, it is not on it’s own a meaningful descriptor.”

We have deleted the text and reference to “medusa” throughout the manuscript, and used the term “alveolar epithelial proliferation” or “pneumocyte type 2 proliferation”.

Figure 6A – quantification of TTF1, IBA1 and MX1 shows no significant differences between BA.1 and BA.5 infected lungs while the text at line 236 says that the BA.5 lungs showed increased expression.

This is a fair point. We modified the text as requested (Lines 283-288):

*“We first investigated BA.5, as other studies, although carried out with chimeric BA.2/BA.5 viruses, suggested that this variant had an increased virulence compared to BA.1 [22] (Fig. 6A, B). Lungs collected from hamsters infected with BA.5 showed in comparison to those infected with BA.1, a consistent trend of increased values for (i) macrophage infiltrate (IBA-1^+^ cells), (ii) cells expressing MX-1 and (iii) alveolar epithelial hyperplasia. Differences however lacked statistical significance (Fig. 6A)”.*

Was a specific lung orientation used for the cuts used in histopathological analysis? How much might different orientations impact the amount of airway vs alveolar space available to contribute to the alveolar epithelial hyperplasia that is measured since this is represented as percent positive area?

Yes, all lungs were embedded and cut in the same way for each experiment. We clarified this point in the Materials and Methods (lines 516-519)

*“We ensured that lungs were always placed in the cassette in the same orientation before the embedding process. Whole organ horizontal sections were also cut at the same level for each animal*.”

In addition, we also compared data of TTF1 quantification obtained from two different lung sections (approximately 100μm apart) of each animal experimentally infected with either BA.1, BA.2 or BA.2.75. We compared the data obtained with both sections (termed for simplicity “section 1” and “section 2”). Data obtained with either of the two sections were very similar and therefore we used a single section per animal in any other experiment. We provided these data as Fig. S7 and referred to this point in the Materials and Methods section (lines 596-601).

General/Line 303 – How was type II pneumocyte hyperplasia selected as the major histopathological phenotype to assess coronavirus pathogenesis? Was TTF1 staining compared to any other markers like Annexin-V for necrosis or others?

As mentioned already in the Discussion (lines 365-368) several studies have already highlighted that alveolar epithelial hyperplasia is a key feature of lesions induced by SARS-CoV-2 in experimentally infected hamsters [29, 41, 42], and it was also found by us and others, in post-mortem samples of human patients dying as result of COVID-19 [51-53] [58]. Note that we added in the revised version the reference to one of our studies currently in pre-print [58]. It is also important to stress, as we did in the discussion (lines 371-377), that we found that infiltrating macrophages and proliferating epithelial cells constitute most of the cells in the affected areas of the lung parenchyma of hamsters infected with virulent SARS-CoV-2 variants. To better illustrate the lung histopathology and the presence of macrophages and type-2-pneumocytes in the majority of the lesions, we added micrographs of immunohistochemistry from serial sections in **Figure S4 F-H**.

In addition, we had also carried out experiments with Caspase-3, to measure and quantify apoptotic cells but we found the number of positive cells too small to be used with confidence. We have added these data below for the perusal of the Editor/reviewers but we don’t think that they are suitable in their current form to be added in the manuscript.

Many more details are needed about the AI training that was done select alveolar epithelial hyperplasia. “Several” slides were used to train the HALO algorithm, how many lungs from different infection conditions? Was this training confirmed for accuracy by a pathologist? Are there scripts that can be shared with other laboratories? Without a standardized, tested and sharable methodology there is no advantage to this process.

We agree with the reviewer. First of all, we would like to confirm that all the histological and image analyses of this study were led by the first co-author and experimental pathologist Vanessa Herder, who is a Diplomate of the European College of Veterinary Pathologists and German *Fachtierarzt fuer Pathologie*. Vanessa has more than 16 years of experience in pathology working with animal models and image analysis. We have added in the Materials and Methods a (i) detailed explanation of all the digital pathology methods (both the simple quantification of signals and the machine-learning assisted imaging) (Lines 535-601) (ii) the algorithm used to reproduce our method (Supplementary Data 1-3), and (iii) an additional Supplementary Figure (Fig. S6) providing examples of the performance of the software.

Reviewer #3: 1) It is problematic to mix male and female hamsters when the virulence of different virus strains is being assessed because it is well known that the sex and age of the hamsters may significantly affect the disease phenotype even when using the exact same virus strain. The authors must either show male and female as indicated by different symbols in each relevant figure, or they should supplement additional experimental data using the same sex (preferably male because the disease tends to be more severe in male hamsters and therefore the phenotypic differences would be easier to observe).

We think it is an excellent suggestion to modify the figures in order to highlight differences between males (now triangles) and females (circles). The figures are now more informative.

There is some evidence in the literature, as the reviewers points out, that male hamsters may develop a more severe SARS-CoV-2-associated disease than females. From the figures of this study, in some cases these differences are substantiated, and males seem to appear slightly more susceptible (e.g. Delta Fig. 5C, 6A; BA.5 Fig 6A; EG.5.1 Fig. 6J-L). Others however don’t seem to show much of a difference (e.g. Omicron Fig. 5B, 6A; XBB Fig 6D, BA.2 Fig. 6G, BA.2.75 Fig. 6G and H), while in one case females seem to be more susceptible (BA.2.75, Fig. 6D, E).

Overall, given the collective data from the literature and our own, there seem to be a slight difference in susceptibility between males and female hamsters, but it is debatable whether in order to phenotype any given variant it is “better” to infect only one gender. Indeed, in the UK we are pushed by our funders exactly in the opposite direction. The argument posed by the funders is that a sex and gender-informed perspective is essential in reporting scientifically accurate and robust data as well starting a research culture in which both genders are reflected ([https://www.ukri.org/councils/mrc/guidance-for-applicants/policies-and-guidance-for-researchers/sex-in-experimental-design/](https://eur03.safelinks.protection.outlook.com/?url=https%3A%2F%2Fwww.ukri.org%2Fcouncils%2Fmrc%2Fguidance-for-applicants%2Fpolicies-and-guidance-for-researchers%2Fsex-in-experimental-design%2F&data=05%7C01%7CVanessa.Herder%40glasgow.ac.uk%7C4d3b841fbb984d3fd67a08dbbe805634%7C6e725c29763a4f5081f22e254f0133c8%7C1%7C0%7C638313230627600071%7CUnknown%7CTWFpbGZsb3d8eyJWIjoiMC4wLjAwMDAiLCJQIjoiV2luMzIiLCJBTiI6Ik1haWwiLCJXVCI6Mn0%3D%7C3000%7C%7C%7C&sdata=bohihUEvKUHCo0Yb%2FynZM%2FXOMufd9ArR0nAk3e5pOFQ%3D&reserved=0)). There are several studies advocating that by not using male and female animals in studies simultaneously, the results of the study are limited to only half of the population (*doi: 10.33549/physiolres.934593; doi: 10.5483/BMBRep.2018.51.4.034*). Our conclusion, after several discussions with many colleagues, is that if there was a very strong difference in the phenotype between males and females on any given assays, we could use only males in order to justify the use of a smaller number of animals. However, if differences are small, it is probably better in the long run to use both sexes, for the reasons highlighted by the publications and guidelines indicated above.

It is important also to stress that our manuscript focuses on the development of a digital pathology pipeline – and this pipeline can be equally applied to experiments designed with only males or males and females.

2) As a key part of this study is the development and validation of the digital pathology assessment pipeline, the same sample sections should be thoroughly assessed by at least 2 well-trained pathologists (who should be unaware of the digital pathology assessment’s findings and scores) as a “control gold standard” of the existing way to assess histopathological damage in the COVID-19 Syrian hamster model.

As mentioned above, we had not stressed that the lead author of this paper is a Board Certified Veterinary Pathologist. For completeness, and to satisfy the reviewer’s suggestion, we have also asked three additional board-certified pathologists to assess one of our experiments (the one comparing BA.1, XBB, BQ.1.18 and BA.2.75; Fig. 6 C-E) using a scoring system as published by Hoagland *et al* (Immunity. 2021 Mar 9;54(3):557-570.e5. doi: 10.1016/j.immuni.2021.01.017; (although we used three pathologists instead of the one used in the study). We have added the results obtained in a supplementary figure (Fig. S8).

To summarise this specific scoring system (for the benefit of the editors/reviewers), this is based on HE-staining of lung sections that are semi-quantitively scored on the bases of the amounts of lung affected, perivascular inflammation, inflamed bronchi, inflamed alveoli, and type 2 hyperplasia.

Please note that other studies look at slightly different histopathological criteria and use for example scores from 0 to 3 (negative), 1 (weak), 2 (moderate) and 3 (severe) (Suzuki, *et al*. *Nature* **603**, 700–705 (2022). https://doi.org/10.1038/s41586-022-04462-1).

Where we respectfully disagree with the reviewer is that the score provided by two individuals represents a “gold standard”. We appreciate the complexities, and we are not critical at all about the approach, which has been the cornerstone of histopathology evaluation for decades. It is simply that this cannot be defined as a “standard”. Scores will vary between individuals, histopathological criteria, and therefore observations are not really quantitative.

We agree that in practical terms within a single study this makes little difference. As shown in Fig. S8 a similar trend of data was obtained in that particular experiment using the digital pathology and the cumulative semi-quantitative score provided by veterinary pathologists in a blind fashion. We simply suggest that a digital pathology pipeline (that of course can be further improved, refined and modified with time) offers a more robust quantitative method, it is faster, and offer the opportunity to compare better results between different laboratories.

3) As correctly mentioned by the authors, a number of histopathology scoring systems for assessing SARS-CoV-2-induced respiratory tract tissue damage have been used in the field. Please add these scores either to the corresponding main figures or as supplementary figures.

As mentioned above, we have provided a side-by-side example from one of the experimental series. We don’t feel however that it would be useful to repeat this side-by-side comparisons for all the experiments. As discussed, it is very difficult to define a standard when this is based on a scoring system based on judgement of individuals.

We appreciate that data may look in a single study less variable if lesions are recorded only in male hamsters and with pathological scores provided by individuals that can only be 0, 1, 2, 3. Indeed, the literature clearly shows statistically significant results with only 4 or 5 hamsters per group using this method. The data from the digital pathology pipeline proposed in this study shows more variability. We believe however that this variability reflects more faithfully the individual variability to SARS-CoV-2 infection. On the other hand, it is also true that that the pipeline proposed may require a larger number of animals to unveil significant differences between variants with differences in virulence that are relatively small.

Part III – Minor Issues: Editorial and Data Presentation Modifications

Reviewer #1: Figure 1 D and E – the gene names are too small to read

The font size has been increased for better readability.

Figure S2 – what is the timepoint of these samples?

We added the information of 2dpi in the text of the revised version and mentioned it additionally in the figure legend.

Figure S3 shows differences in spike RNA in turbinates and trachea only and no significant differences in Mx1 expression. Please modify the text at line 188 to reflect the data.

We rephrased the text accordingly in the revised manuscript (Lines 234-240): *“The differences between Delta- and BA.1-infected hamsters were also present at earlier timepoints. Analysis of tissues collected at 1 dpi of the turbinates, trachea, and lungs (Fig. S3A) showed increased levels of spike RNA (Fig. S3B) in Delta-infected animals. MX1 expression showed a trend of higher levels of expression in Delta-infected animals but differences were not statistically significant (Fig. S3C). Similarly, nasal washes whole lung and throat swabs showed higher levels of genomic RNA in Delta-infected hamsters, but only in the latter differences were statistically significant (Fig. S3D).*

Line 182 – description of Figure 3 should mention the 2 DPI timepoint

We added the information in the text.

Figure 3 – the lung staining images shown do not seem representative of the quantitation for Mx1 or IFIT1

We apologise, it was an oversight on our part to not correctly label the Y axes. This has been changed now and the values are given in percentages of pixel positive per total pixels detected in the entire lung section. We also added in the Methods, the full description of how these data are obtained (Lines 535-561).

Figure 4 – BA1 sections should show large airways for effective comparison with mock and Delta images.

We replaced the sections and have now included photos showing large airways in all panels in figure 4.

Line 215 – please add at 6 dpi.

We have added 6 dpi as requested.

Figure 5 C – please explain the 3 panels, in particular what is the left most lung section?

Figure 5D – Are these panels in same samples/order as 5A and B?

We thank the reviewer. We have changed the figure and the legend to make it clearer to the reader.

Pax5 staining is mentioned in the materials and methods but does not seem to appear in the manuscript?

The reviewer was right. However, we have now used this stain in one of the supplementary figures in order to address a concern of the reviewer. Hence, the description of the PAX5 stain has now been left in the M&M section.

Line 262 refers to figure 6G, not 6H. 6H should be discussed in the text.

We made the required changes and also highlighted for 6H that all variants induced limited levels of alveolar epithelial hyperplasia (Fig. 6H), in line with the limited amount of virus replication in the lungs.

Line 310 – Did the authors demonstrate anywhere that infiltrating macrophages and proliferating epithelial cells make up most of the cells in the lung parenchyma? Neutrophils are an important and abundant cell type in the lung after coronavirus infection and should also be quantitated to allow for this statement. This would be an important supplemental figure to add to justify IBA1 and TTF1 staining as key markers of pathogenesis.

We included a new supplementary Figure (Figure S4) where we show by H&E infiltration with neutrophils/heterophils in the lungs of hamsters infected with the Delta variant. Inflammatory lesions are also characterised by the presence of lymphocytes and plasma cells (as shown also in the figure with CD3 and PAX5 Abs in IHC. Overall, however, IBA1^+^ and proliferating type 2 pneumocytes (showed by TTF1 and E-Cadherin staining in Fig S4) appear to be the most abundant cells in the pulmonary lesions induced by Delta.

We are not aware of a validated Ab for neutrophils in the hamster. We have tried a neutrophil-specific Ab that shows reactivity with the right cell types but as it can be seen on the left, reactive cells are not as abundant as macrophages and therefore would not add significantly to the digital pathology pipeline developed.

Line 150 vs Line 636 – one describes nucleocapsid IHC for figure S2 and one describes spike IHC, please clarify which is correct

We detected with the IHC the nucleocapsid protein and the spike by in situ-hybridisation. We corrected this in the entire manuscript.

Line 289 – The host immune response is also dictated by animal welfare and wellbeing prior to infection. These statements seem overly critical of WL and especially respiratory function as measures of disease.

This is a fair point. We have deleted that sentence and highlighted that the key advantage of the digital pipeline proposed is the opportunity to more accurately quantify the pulmonary lesions (lines 349-357, 373-381, 430-424).

Figure 6 – Please clarify in the text and figure legend that the TTF1 expression being quantitated is the software selected alveolar regions.

We rephrased this part of the text and, as mentioned above, added in-depth details that we followed in the Methods (lines 562-601). The algorithm was tuned to detect pneumocyte type 2 hyperplasia in the alveolar regions and ignoring hyperplastic as well as normal bronchial epithelium. For a better illustration, we also included an additional supplementary figure (Fig. S6) showing the performance of the final algorithm.

Reviewer #2: Line 385 in the cells and viruses. The authors clarified that the viruses were isolated from swab samples. The authors should then clarify in detail how the virus was purified to avoid any other viruses. For example, the authors may conduct PCR to detect other common respiratory viruses such as RSV, influenza, rhinovirus, etc. The authors may also do several rounds of serial dilution after virus passage before conducting PCR.

This is normally not an issue. Neither human influenza viruses, nor RSV, nor rhinoviruses infect hamsters readily. In addition, we quantify viruses by PCR (using the same amount of genomes) to avoid biases on how different variants replicate in different cell types. We prefer to use virus isolated minimally in tissue culture as it better represent the clinical isolates. Extensive passage in cell culture can lead to in vitro adaptation and the emergence of spurious mutations.

Line 576, legend for figure 4. I don't think the histopathology upon SARS-CoV-2 infection is equally distributed in the lung, unless there are papers describing it otherwise. So it is optimal to show the tissue from the same lung lobe at each timepoint.

We updated figure 4 and included always the same lobe in the pictures.

Figures. The figures often use "positive area" as a parameter. However, in the methods section, it was not clear how the positive area was determined. In figure 3 for example, the background of D and E seems higher for both Delta and BA1, is it the reason their positive areas are bigger than A and B?

As mentioned above, we corrected this throughout the entire manuscript, updated the Methods section and changed the figure legends accordingly (lines 535-601).

Reviewer #3: 1) Figures: The corresponding viral loads should be provided to allow easier correlation between histopathological damage, clinical scores, and viral burden in the figures.

We have added the information about the virus load used in the experimental infections in each relevant figure legend. We prefer to leave the PCR data in the supplementary figure to leave the main figures focused on the pathology.

2) Please make sure to spell out the full name of each terminology when it is used for the first time in the manuscript (eg: COVID-19 in line 45).

Changes have been made in the entire manuscript.

3) Lines 382-385: Please state explicitly whether the virus strains were plaque purified to avoid having mixed strains in the same sample.

No, we don’t plaque purify the virus to avoid infecting with a single plaque that is not representative of the clinical isolate. However, virus isolates were deep sequenced and mixed variants would be picked up at that stage.

We thank again all the reviewers for their insight, and we trust that the manuscript is now ready for publication.

Your Sincerely

**Massimo Palmarini**
